# Non-aqueous green solvents improve alpha-amylase induced fiber opening in leather processing

**DOI:** 10.1038/s41598-020-79406-8

**Published:** 2020-12-17

**Authors:** Poornima Ramamoorthi, Aravindhan Rathinam, Raghava Rao Jonnalagadda, Thanikaivelan Palanisamy

**Affiliations:** 1 Leather Processing Technology Division, CSIR-Central Leather Research Institute, Adyar, Chennai 600020 India; 2 Inorganic and Physical Chemistry Laboratory, CSIR-Central Leather Research Institute, Adyar, Chennai 600020 India; 3 Advanced Materials Laboratory, CSIR-Central Leather Research Institute, Adyar, Chennai 600020 India

**Keywords:** Biotechnology, Biocatalysis, Green chemistry, Process chemistry

## Abstract

Severe water deficit and highly polluting effluent generation from leather industries have constantly been pressurizing the tanners to adopt cleaner leather processing systems. The present study aims to minimize the use of water by substituting it with non-aqueous green solvents and also to enhance the enzyme action in alpha-amylase based fiber opening process. The activity of alpha-amylase in select non-aqueous green solvents namely, heptane, polyethylene glycol 200 and propylene glycol is considerably higher by 62, 38 and 31% than in water, respectively. Comparable results are obtained for the catalytic efficiency of alpha-amylase and hence it is further validated in collagen fiber opening trials as well. Scanning electron micrographs, histological images and proteoglycan estimation supported the above findings at 1% alpha-amylase dosage. The final quality of the experimental leathers in terms of physical and bulk properties is comparable to that of control leathers. Recycling studies indicate that it is possible to replace water with green solvents for enzymatic fiber opening with the feasibility to recover more than 85% solvent-enzyme mixture and reuse without any additional alpha-amylase usage. Reduction in pollution load coupled with the efficient catalytic action of enzyme in non-aqueous media favors the present protocol for industrial applications.

## Introduction

Leather industry is an offshoot of the meat industry since the skins and hides are predominantly supplied from the meat industry; it also has economic relevance due to the offering of various consumer products^1,2^. Traditional leather processing involves various unit processes and operations that require intensive use of chemicals and water. These processes employ nearly 25–40 L water per kilogram of skins^3^. Increasing global concerns for environmental pollution and water scarcity has led to a negative impact on the tanneries. Resolving the issue of pollution coupled with operation, maintenance and treatment costs remain as a serious challenge for tanners all over the world. Conventional dehairing and fiber opening processes utilize lime and sodium sulfide along with water, which in turn release effluent with about 60–70% of total pollution load^4–6^. Though lime itself is not toxic, large amount of sludge produced makes it an issue in safe disposal. Emission of toxic gases like hydrogen sulphide as a result of mixing of dehairing and pickling streams is another major challenge for the tanners^7,8^.

Advances in enzyme technology have led to the use of various enzymes in leather manufacture in soaking, dehairing, bating and degreasing processes^9^. Although several enzymatic dehairing and fiber opening methods have been developed, many of these processes have not yet been commercialized^1,10,11^. However, enzyme assisted dehairing with low amount of sodium sulfide is being followed in some parts of the world. Alpha-amylase or NaOH based fiber opening has been standardized for cow hides and goat skins^10,12,13^. However, reduced efficiency and the strict process control measures in enzyme-based fiber opening limits its commercial exploitation so far. Attempts have also been made to integrate the two-step enzymatic processes such as enzymatic dehairing using protease and fiber opening with alpha-amylase into a single-step process by successive and simultaneous enzyme application^14,15^.

Typical enzyme catalysis is carried out in aqueous systems, and it was believed that enzymes are active only in water until studies on non-aqueous enzymology has been attempted at the end of twentieth century^16–19^. Initial studies on non-aqueous enzymology were carried out using water-miscible organic solvents like ethanol, which was then replaced with biphasic mixtures. Attempts have been made to carry out reactions in nearly non-aqueous solvents containing trace amounts of water^20–22^. The role of water in catalysis is critical as it influences the biomolecular structure, function, and interactions^23,24^. The key determinant of enzymatic activity is the amount of hydration water or enzyme-bound water available for the solvation of the enzyme^16^, which is needed in trace amounts as low as 0.1%. Hydrolytic enzymes like protease and lipase have been shown to carry out reactions exhibiting change in selectivity, pH memory and increased activity and stability in non-aqueous solvents^19,23,25,26^. Castro et al. have studied the change in the activity of few proteases in neat organic solvents based on the cleavage of bovine serum albumin (BSA) by the enzymes^27^. They reported the change in the specificity of the enzyme by switching the solvent from water to glycerol.

The present study aims to understand the ability of alpha-amylase to open up the fiber bundles in the skin matrix in the presence of non-aqueous green solvents used as an alternative to water. GlaxoSmithKline (GSK) periodically releases solvent selection guide to screen and select solvents based on a relative ranking of the inherent environmental, health, safety and life cycle assessment issues associated with each solvent^28^. Based on the green chemistry principles such as polarity, environment and health hazards and LD_50_ values, seven green solvents namely ethanol, ethyl acetate, ethyl lactate, heptane, propylene carbonate, propylene glycol and polyethylene glycol 200 (PEG 200) were screened and selected from the list of solvents suggested by GSK^29–31^. The activity of the enzyme in selected green solvents and water as control has been analyzed and compared. Accordingly, the solvents exhibiting higher or enhanced activity were chosen for application in fiber opening of the skin matrix. The concentration of enzyme and time for fiber opening has been standardized using drum method for sheepskins in the chosen solvents. The extent of fiber opening has been assessed using proteoglycan assay, scanning electron microscopy and histological examination. The experimental and control leathers have been analyzed for bulk and physical properties. The extent of pollution loads in the collected effluent has been quantified. The possibility of recycling enzyme-solvent mixture to the next batch of fiber opening process has also been investigated.

## Materials and methods

### Materials

Wet salted red hair sheepskins were chosen as raw material for the experiments and were procured from a slaughterhouse located at Chennai, India. Commercial grade dehairing enzyme, (Microdep C, protease formulation) was procured from Tex Biosciences Pvt. Ltd. India and commercial grade alpha-amylase (fiber opening enzyme) was procured from United Alacrity India Pvt Ltd., India. All chemicals used for leather processing were of commercial grade. Solvents namely ethanol, ethyl acetate, ethyl lactate, heptane, propylene carbonate, propylene glycol and PEG 200 were of analytical grade and purchased from Loba Chemie, India. All the reagents used for the assay were of analytical grade and purchased from SRL Pvt. Ltd., India.

### Amylase assay in green solvents

Activity of alpha-amylase in the selected green solvents namely ethanol, ethyl acetate, ethyl lactate, heptane, propylene carbonate, propylene glycol and PEG 200 was measured according to the method proposed by Bernfeld employing dinitrosalicylicacid (DNSA) with slight modifications for the solvents^32^. For control assay, water was used instead of the solvent. Starch was used as substrate and the amount of maltose released in the enzyme reaction was measured using a spectrophotometer. The reaction mixture consisted of 1 ml of starch (10 mg/ml in sodium phosphate buffer pH 6.9), 1 ml of the solvent under study and 1 ml of the enzyme solution (50 mg/ml in water) and incubated for 10 min. The enzyme reaction was terminated with the addition of 1 ml DNSA and allowed to boil for 15 min and cooled in cold water bath (4 °C) and made up to 13 ml by adding distilled water. The development of reddish-brown color indicates release of maltose from starch and the absorbance of this solution was recorded at 540 nm using UV–visible spectrophotometer (Hitachi, U-2000). Control for each solvent was also set up with the same procedure without addition of enzyme.

Standard graphs were plotted by varying the maltose concentration in various solvents as shown in Supplementary Fig. S1. The concentration of maltose was varied from 0.4 to 4 mg/ml. Initially, stock solution of maltose with a concentration of 2 mg/ml was prepared in water as it was sparingly soluble in other chosen solvents. Subsequently, varying volumes (0.2–2.0 ml) of the stock solution was made up to 3 ml by adding 1 ml of respective solvent and remaining with water. To this mixture, 1 ml of DNSA was added and allowed to boil for 15 min. Then, the reaction mixture was cooled in cold water bath (4 °C) and made up to 13 ml by adding distilled water. Absorbance of the same was recorded at 540 nm using UV–visible spectrophotometer. As can be seen, the presence of the organic solvents used in this study did not alter the standard line significantly. The deviations in the calculated enzyme activity were within the error range of ± 0.5 mmol/min/ml and hence the standard graph derived using water was used for all further calculations. The enzyme activity as a measure of amount of maltose released was derived from this graph (Supplementary Fig. S1a). One unit of the enzyme is equivalent to the liberation of 1 mg of maltose per ten minutes from starch at pH 6.9 at 30 °C. The end point assay demonstrating the linearity of initial reaction rates over a period of 10 min is given in Supplementary Fig. S2. The enzyme activity was calculated using the following equation.

1 $$Enzyme\, activity\, (mmol/min/ml)=\frac{\left[Maltose\right] \times Dilution \,factor}{Volume\, of\, enzyme \,used \times time\, of\, assay \times MW\, of\, maltose}$$

where [Maltose] is the concentration of maltose obtained from standard graph, mg/ml, dilution factor is the total volume used in the assay, ml, the volume of enzyme used, ml, time of assay is the total time of incubation substrate with the enzyme, min and MW of maltose is the molecular weight of maltose, g/mol. All trials were carried out in triplicate. Those green solvents, which showed enhanced enzyme activity in comparison to aqueous medium, were chosen for further studies.

### Reaction kinetics

The effect of substrate concentration on alpha-amylase activity in the presence of solvents was studied using the procedure stated in the previous section with slight modification. The concentration of starch in the reaction medium was varied in the range of 10 to 100 mg/ml, at the alpha-amylase activity of 4.6 mmol/min/ml in water, 6.5 mmol/min/ml in heptane, 5.3 mmol/min/ml in propylene glycol and 6 mmol/min/ml in PEG 200 and keeping incubation time (10 min) constant, as described in the procedure stated in the previous section. The effect of substrate concentration in the presence of the three non-aqueous solvents namely heptane, propylene glycol, PEG 200, and water (control) was analyzed^33^. Table S1 (supplementary Information) shows the raw data derived using Eq. (1) for calculating the enzyme activity in each green solvent and enzyme–substrate reaction rates in water (control), heptane, propylene glycol and PEG 200. Michaelis–Menten kinetics was assumed for the enzyme–substrate reaction. Michaelis–Menten equation is as follows.

2 $$V=\frac{{V}_{max} \times \mathrm{ S}}{{K}_{m}+\mathrm{ S}}$$

where V is the initial rate (velocity) of the reaction, mmol/min/ml, V_max_ is the maximal rate of the reaction, mmol/min/ml, S is the concentration of the substrate, mg/ml and K_m_ is the Michaelis–Menten constant, mg/ml.

A graph for the Michaelis–Menten equation (Eq. 2) was plotted with the predicted initial reaction rate, V, as a function of the substrate concentration, S, to depict the kinetic parameters V_max_ and K_m_ graphically.

Reciprocal of the Eq. (2) yields to the following equation.

3 $$\frac{1}{V}=\frac{1}{{V}_{max}}+\left[\frac{{K}_{m}}{{V}_{max}}\times \frac{1}{S}\right]$$

Using the substrate concentration data for each solvent, a Lineweaver–Burk plot (1/V vs. 1/S) was drawn to determine the kinetic parameters of the enzyme-assisted reaction, which yielded a straight line with Y-intercept of 1/V_max_ and slope of K_m_/V_max_^34,35^.

Catalytic rate constant or turnover number, K_cat_, for the enzyme as a measure of its ability to catalyze or convert a particular number of substrate molecules per second can be calculated from the V_max_ using the following equation.

4 $${V}_{max}={E}_{o}\times {K}_{cat}$$

where E_o_ is enzyme concentration in mg/ml and K_cat_ is the turnover number in S^–1^.

### Optimization of enzyme concentration for fiber opening in solvents

Four wet salted sheep skins were weighed and soaked with water at the proportion of 3 times their weight for a period of 8 h. After soaking process, the skins were drained of water and the soaked weight of the skins was noted. Subsequently, dehairing was carried out by pasting method, where 2% dehairing enzyme and 10% water (based on soaked weight) were used to prepare the paste. The paste was applied on the flesh side of each skin and the skins were piled flesh to flesh for a period of 18 h. The skins were then dehaired manually using a blunt knife. The dehaired pelts were then subjected to fiber opening using varying concentrations of alpha-amylase [0.25, 0.5, 0.75 and 1% (w/w)] and 100% (v/w) solvent (based on dehaired weight), respectively for each selected solvent namely heptane, propylene glycol and PEG 200 for 1 h. For control, the same trial was carried out with 100% (v/w) water (based on dehaired weight). The change in weight of the pelt after fiber opening for each enzyme concentration in respective solvents and water was noted. The extent of fleshing and grain intactness was also noted.

### Enzymatic fiber opening in selected green solvents (with optimized alpha-amylase concentration)

Twelve salted sheep skins were weighed and soaked with water at the proportion of 3 times their weight for a period of 8 h. After soaking process, the skins were drained of water and the soaked weight of the skins was noted. Dehairing enzyme was applied on the flesh side of the soaked skins and left overnight (18 h) following the procedure as given above. Following day, the skins were dehaired using a blunt knife and the weight was noted. The dehaired pelts were then processed in drums. Each skin was subjected to fiber opening with 1% (w/w) alpha-amylase (optimized concentration) in presence of 100% (v/w) solvent (based on dehaired weight) respectively for each selected solvent namely heptane, propylene glycol and PEG 200 for 1 h^36^. For control, the same trial was carried out with 100% (v/w) water (based on dehaired weight). The experiments were carried out in triplicate. The spent liquor from the experiment and control was collected at the end of fiber opening and was assayed for proteoglycan release as given below. The opened-up skins were then pickled, chrome tanned and post tanned conventionally into crust upper leathers as detailed in Supplementary Table S2. To evaluate the efficiency of fiber opening, the pelts were subjected to morphological and structural analysis as detailed below. The composite liquor was collected and analyzed for pollution load as detailed below. The experimental and control crust leathers were examined for bulk and physical properties as detailed below.

#### Scanning electron microscopic analysis

Samples from control and experimental crust leathers treated with 1% alpha-amylase were cut at the specified positions with uniform thickness^37^. Samples were dried by gradient dehydration method in ethanol gradually. The dried samples were then coated with gold using an Edwards E306 sputter coater. Leica Cambridge Streoscan 440 microscope operated at 20 kV was used for the analysis. Micrographs for surface and cross section of the specimen were obtained with different magnifications.

#### Histological analysis

Samples from control and experimental leathers treated with 1% alpha-amylase were subjected to Hematoxylin & Eosin (H&E) staining protocol to study the degree of fiber opening. The samples were cut from opened up pelts without any pre-treatment from the specified sampling position^37^. The samples were then coated with paraffin from which thin sections were cut and mounted on a microscopic slide for staining. The stained slides were examined under compound optical microscope^38^.

#### Analysis of composite liquor

Composite liquors from experimental and control processes were collected after each unit process excluding soaking until tanning. The liquor was analyzed for total dissolved solids (TDS) in effluent^39^. Emission loads were calculated by multiplying concentration (mg/L) with volume of effluent (L) per metric ton (t) of raw skins processed.

#### Shrinkage temperature (Ts) of control and experimental leathers

The shrinkage temperature of experimental and control wet blue leathers was analyzed using a shrinkage tester, SATRA STD 114 apparatus, with samples of 1 × 2 cm^2^. Samples were cut from the wet blue leathers at the official sampling position as per the IUP method^37^. Samples were then suspended in the shrinkage tester filled with water and the instrument was heated externally using a burner under closed pressure until shrinking of the sample leather was visually observed. The temperature at which the leather shrinks was recorded as the shrinkage temperature.

#### Physical and bulk properties of leathers

For carrying out the characterization of physical properties, samples from experimental and control crust leathers were cut from the official sampling position as per IUP method^37^. The leathers were conditioned for 48 h at 20 ± 2 °C and 65 ± 2% relative humidity. Physical properties namely tensile strength, tear strength, elongation at break and grain crack strength were determined as per standard procedures^40–42^. Various organoleptic properties such as softness, fullness, grain smoothness, grain tightness and general appearance of the experimental and control crust leathers were rated by three expert tanners on a 0–10 scale by hand and visual examination. The average of the rating from three expert tanners corresponding to each functional property was calculated along with the standard error. Higher ratings denote better property.

#### Reusability of the spent solvent-enzyme mixture

Reusing ability of the spent solvent-enzyme mixture was studied by using spent mixture for a consecutive trial of fiber opening process. Reusability of heptane was given preference as it is volatile compared to the other two solvents. Four wet salted skins were cut into halves and each half was subjected to fiber opening consecutively in the same bath for same interval as that of the standardized time. The spent liquor was assayed for proteoglycan release as detailed below. Activity of alpha-amylase in the spent liquor was also assayed as detailed above for determining the additional dosage requirement for the subsequent reuse. Further studies on consecutive reusability of the spent liquor for all three selected solvents were carried out. Four wet salted skins of approximately same weight were subjected to fiber opening in the used solvent-enzyme mixture in each selected solvent without any additional enzyme use. Another study was carried out to examine the stability of the used solvent-enzyme mixture by ageing for 30 days by measuring the activity of alpha-amylase as described above.

#### Quantification of proteoglycan release

Spent liquor from the standardized fiber opening process carried out in selected green solvents and water medium was collected. The liquor was filtered and used for estimation of proteoglycan using Schiff’s assay^43,44^. Mucin (glycoprotein) was used as the standard. To a set of 10-50 µg of mucin standards in 1 ml of water, 100 µl of freshly prepared periodic acid solution (a mixture of 10 ml of 7% acetic acid and 10 µl of 50% periodic acid) was added and incubated at 37 °C for 2 h. 100 µl of decolorized Schiff’s reagent was added and allowed for color development at room temperature for 30 min. The absorbance at 555 nm was recorded using a Hitachi U-2000 UV–Visible spectrophotometer. Water was used as blank. The standard curve was plotted between mucin standard concentrations against absorbance at 555 nm. The amount of proteoglycan present in the sample was determined from the standard graph.

### Ethical approval

This article does not contain any studies with animals performed by any of the authors.

## Results and discussion

### Enzyme activity in organic solvents

**Figure 1:**
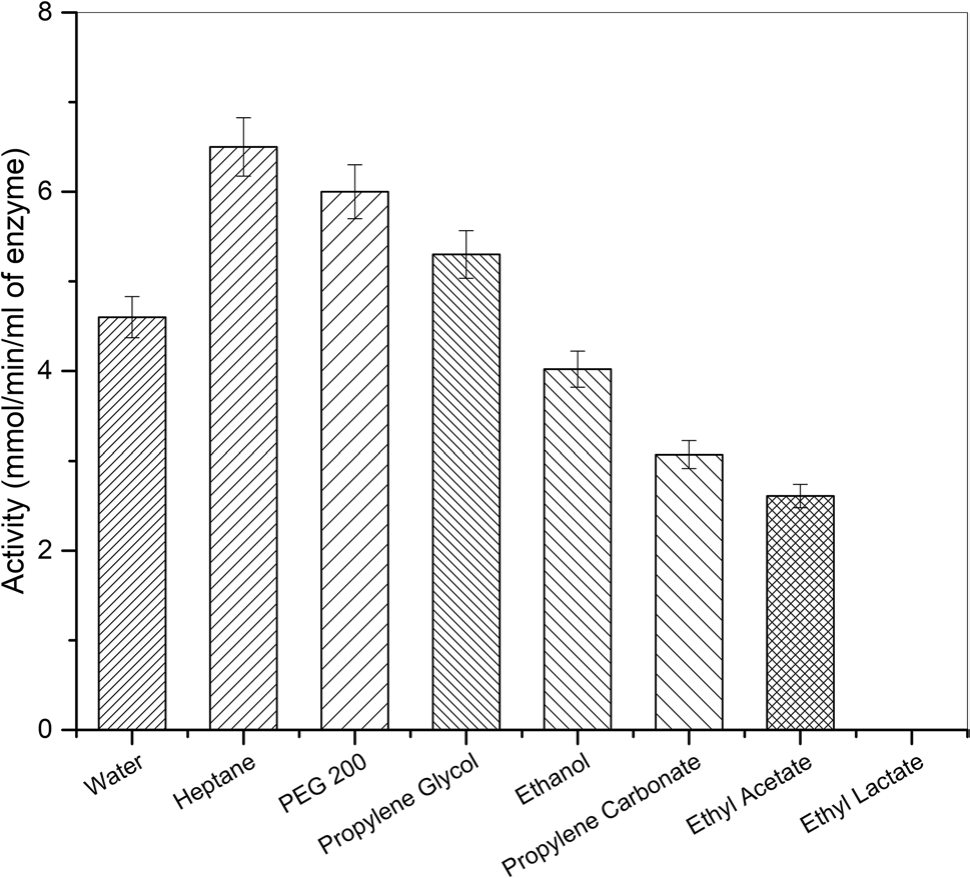
Activity of alpha-amylase in various solvents analysed by employing 1 ml each starch (10 mg/ml) and enzyme (50 mg/ml) solutions incubating at 30 °C at pH 6.9 for 10 min.

The alpha-amylase at a concentration of 50 mg/ml in water was assayed in presence of the non-aqueous green solvents and the activity in terms of mmol/min/ml using DNSA assay was recorded. Figure 1 shows the activity of alpha-amylase in different solvents as well as in water. It is interesting to see that the enzyme activity is highest in heptane medium rather than in water. Indeed, both PEG 200 and propylene glycol exhibit enhanced activity in comparison to the aqueous medium. On the other hand, ethyl lactate completely inhibited alpha-amylase. The activity of alpha-amylase in presence of different green solvents can be ordered as Heptane > PEG 200 > Propylene glycol > Ethanol > Propylene carbonate > Ethyl acetate > Ethyl lactate. This tendency of enhancing the activity of the enzyme in certain solvents can be attributed to their low dielectric and hydrophobic nature. Water plays an essential role in bio-catalysis. The water surrounding the active site of the enzyme molecule confers flexibility in solution. It is known as hydrated or bound water. In the presence of heptane, a hydrophobic solvent, event of stripping of the hydration water around the enzyme’s active site will not take place unlike a water-miscible solvent^45,46^. Enzyme in organic solvents becomes rigid due to lack of water for its conformational mobility and ability to engage in hydrogen bond formation thereby leading to strong intra-protein electrostatic interaction^47^. In such cases, the enzyme activity can be enhanced by adding trace amounts of water or the addition of solvents capable of forming multiple hydrogen bonds^48^. In the case of PEG 200 and propylene glycol having multiple OH groups, the increase in enzyme activity can be inferred with the fact that they are capable of forming hydrogen bonds and can mimic the effect of water^46^. On the other hand, heptane due to its extremely low dielectric constant should make the enzyme rigid due to its inability to engage in hydrogen bond formation. However, the alpha-amylase assay contributed a small amount of water (2 ml) used for preparing starch and enzyme solution, which helped in the conformational mobility of the alpha-amylase and resulted in the enhanced (highest) activity for heptane in comparison to any of the studied solvents including water. In the case of ethyl lactate, it is reported that the pure ethyl lactate exhibits strong intermolecular association competing with the intramolecular hydrogen bonding of water^49^. This behavior is expected to strip off the hydration water around the enzyme’s active site leading to complete inhibition as observed here. These results are in agreement with our earlier report on the protease-green solvents interactions^50^. The three green solvents namely, heptane, propylene glycol and PEG 200, which resulted in higher activity in comparison to water, were chosen for further experiments.

### Enzyme kinetics study

**Figure 2:**
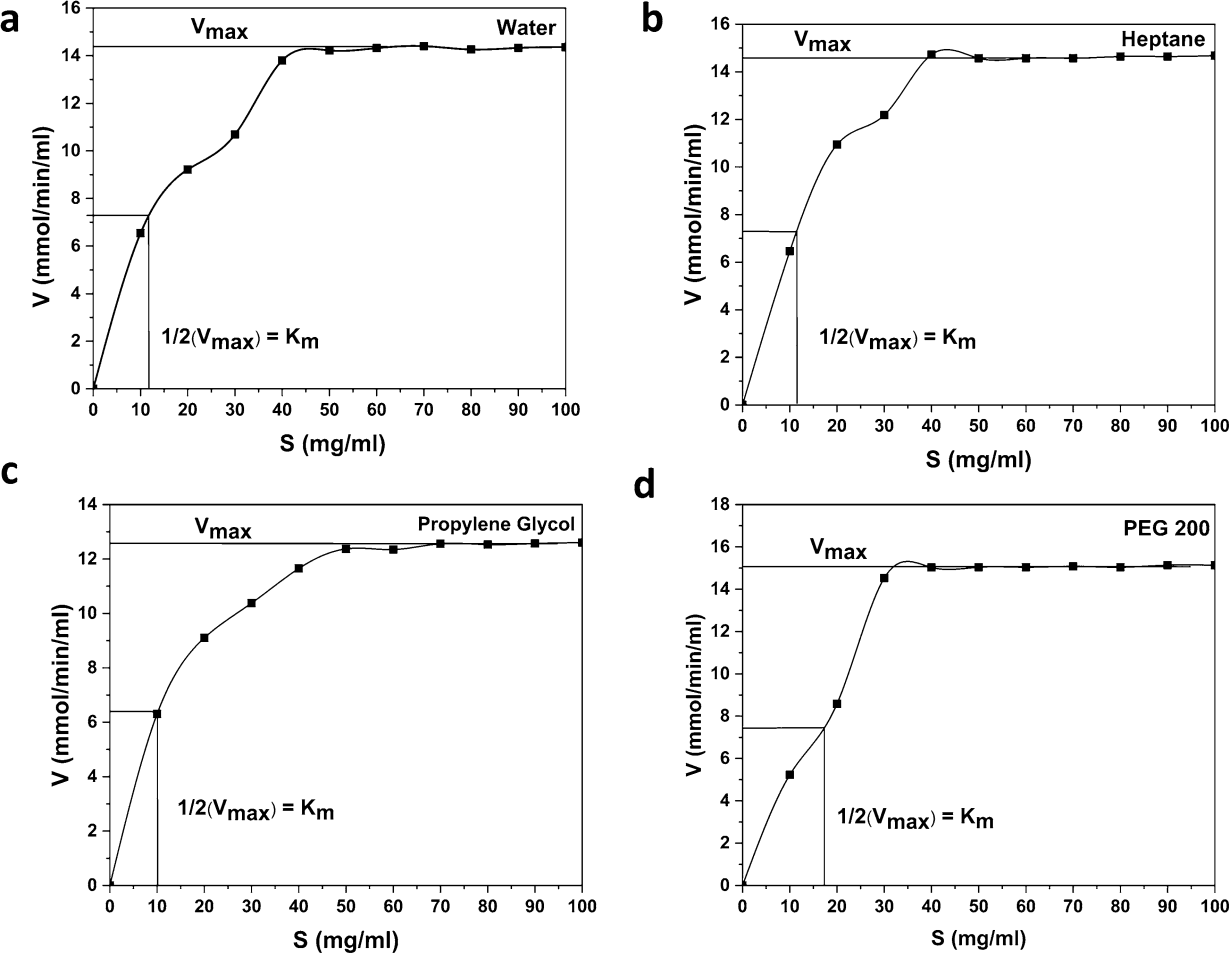
Michaelis–Menten plot of the enzyme substrate reaction (V vs S) (**a**) Control (water), (**b**) Heptane, (**c**) Propylene glycol and (**d**) PEG 200. Reaction was conducted by varying the concentration of starch in the range of 10–100 mg/ml along with 1 ml of enzyme (having activity of 4.6 mmol/min/ml in water, 6.5 mmol/min/ml in heptane, 5.3 mmol/min/ml in propylene glycol and 6 mmol/min/ml in PEG 200) and 1 ml of the respective solvent incubating at 30 °C and at a pH of 6.9 for 10 min.

**Figure 3:**
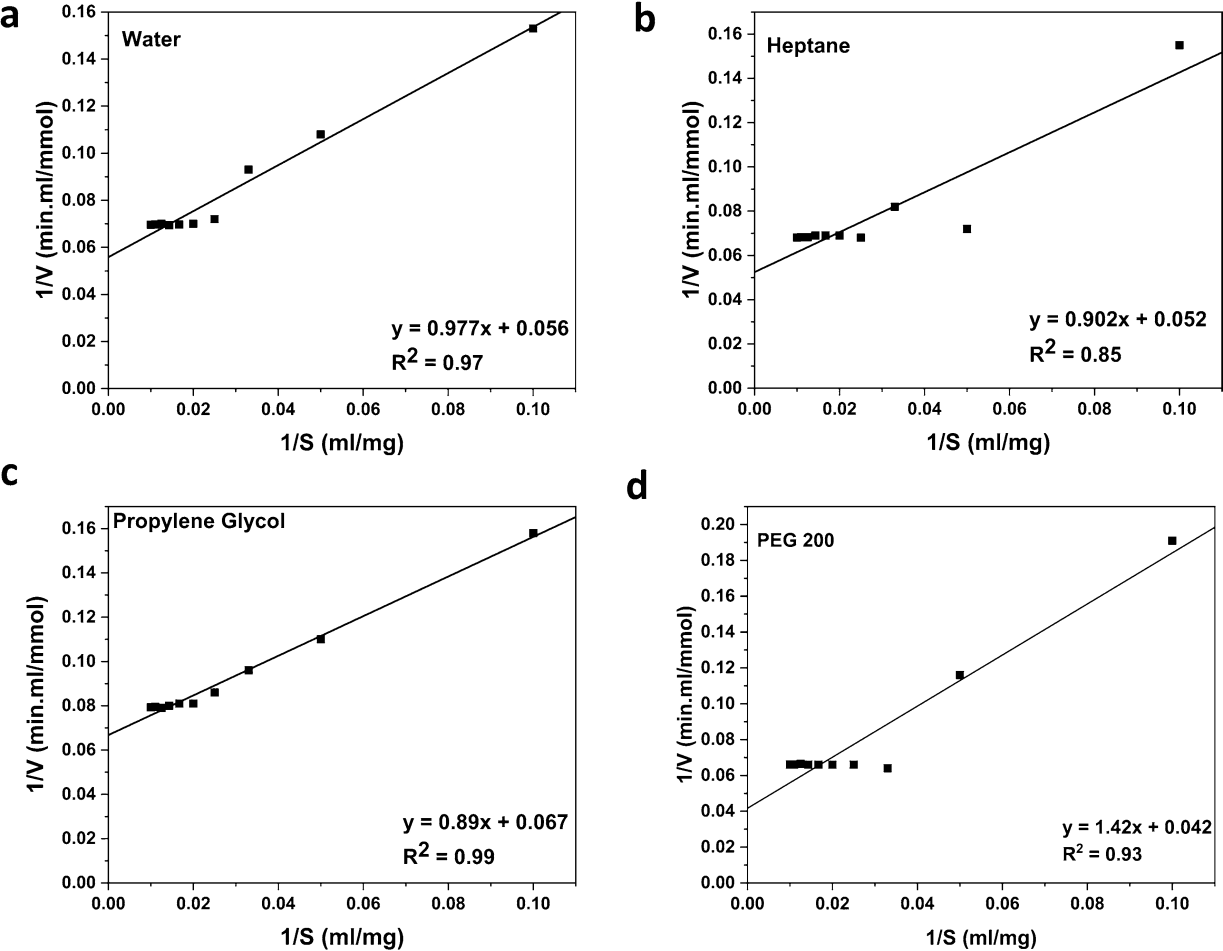
Lineweaver–Burk plot for enzyme–substrate reaction (1/V vs 1/S) (**a**) Control (water), (**b**) Heptane, (**c**) Propylene glycol and (**d**) PEG 200. Reaction was conducted by varying the concentration of starch in the range of 10–100 mg/ml along with 1 ml of enzyme (having activity of 4.6 mmol/min/ml in water, 6.5 mmol/min/ml in heptane, 5.3 mmol/min/ml in propylene glycol and 6 mmol/min/ml in PEG 200) and 1 ml of the respective solvent incubating at 30℃ and at a pH of 6.9 for 10 min.

The Michaelis–Menten plots (V (mmol/min/ml) vs. S (mg/ml)) of the enzyme–substrate reaction in each solvent namely water, heptane, propylene glycol and PEG 200 are presented in Fig. 2 (a–d). The Michaelis–Menten plots show a saturation plateau as the starch concentration increases beyond 50 mg/ml for water and the selected solvents. This means that the available enzyme molecules in the reaction are saturated with the substrate concentration of 50 mg/ml and increasing the substrate concentration beyond this will not increase the maximum reaction rate (product generation rate). From the Michaelis–Menten plots, the maximum reaction rate, V_max_, and the Michaelis–Menten constant, K_m_, can be predicted. However, the Lineweaver–Burk plot (1/V vs. 1/S) was employed for obtaining the values of V_max_ and K_m_ (kinetic parameters) as linearizing the data increases the accuracy. Figure 3 (a–d) shows the Lineweaver–Burk plots for the enzyme–substrate reaction in water, heptane, propylene glycol and PEG 200 medium, respectively. The V_max_ (mmol/min/ml) and K_m_ (mg/ml) were found to be 19 and 18.18 for heptane, 23.9 and 34.5 for PEG 200, 14.9 and 13.7 for propylene glycol and 17.86 and 18.18 for water, respectively. The K_cat_ value for heptane, PEG 200, propylene glycol and water were determined to be 8, 10, 6 and 7 S^–1^, respectively. Higher K_cat_ indicates a higher turnover of the substrate. A lower K_m_ indicates that low substrate concentration is sufficient to reach half maximum reaction rate (V_max_)^51^. The K_cat_/K_m_ value provides the efficiency of the enzyme both in terms of better affinity for the substrate as well as a fast reaction rate. The catalytic efficiency of the enzyme in heptane, PEG 200, propylene glycol and water were found to be 0.44, 0.29, 0.43 and 0.38 respectively. It is evident that heptane and propylene glycol medium show slightly higher catalytic efficiency compared to water and provide higher affinity for the enzyme to the substrate with a fast reaction rate thereby suggesting that the enzyme reaction is feasible even in hydrophobic solvent. The kinetics data support that the selected green solvents, excluding PEG 200, can efficiently replace water. Although PEG 200 showed higher enzyme activity than water (Fig. 1), it is seen that the reaction kinetics are slightly inferior to water medium.

### Optimization of enzymatic fiber opening in green solvents

**Figure 4:**
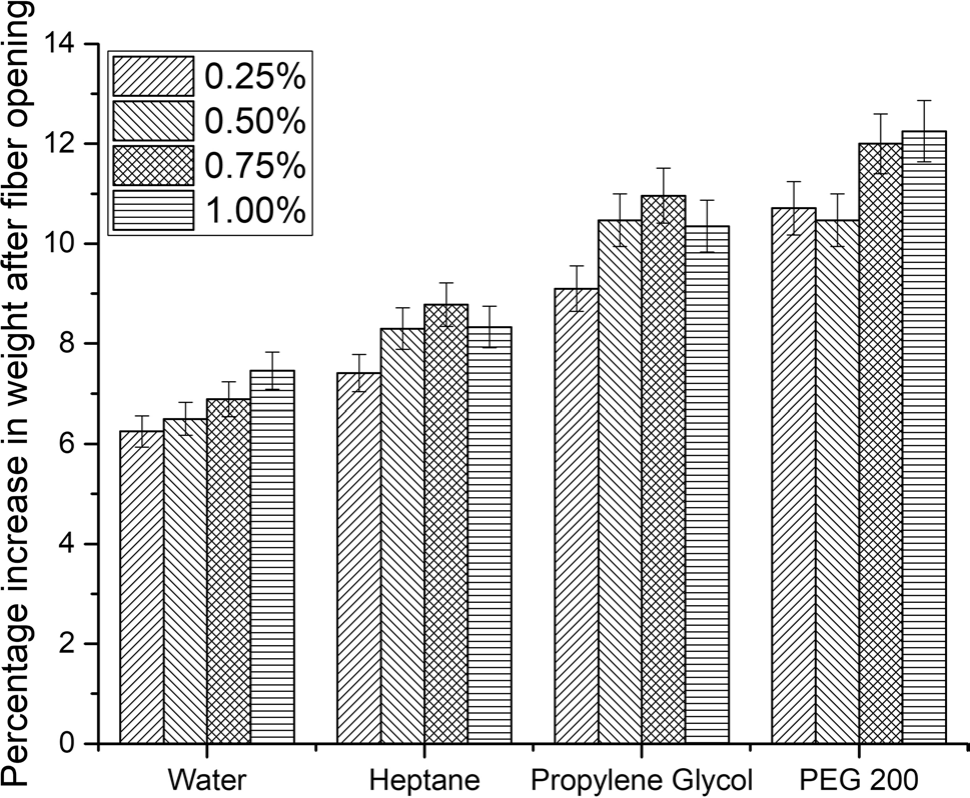
Percentage increase in weight of the skins after fiber opening in various solvents (before fleshing).

Enzyme concentration was varied as 0.25, 0.5, 0.75 and 1.0% (w/w) with 100% (v/w) green solvent for experiments and 100% (v/w) water for control. The extent of fiber opening in terms of percentage increase in pre-fleshed weight to dehaired weight was compared. Figure 4 shows the percentage increase in pre-fleshed weight of the skins after fiber opening for each solvent and enzyme concentration. It is seen that the swelling (increase in pre-fleshed weight) for all three solvents and water is in the range of 9 ± 3% up to 0.5% alpha-amylase. Higher concentrations of the enzyme (0.75 and 1%) produced better swelling in all three solvents (10.5 ± 1.5%), more specifically in PEG 200, than in water medium. The inherent water present in the skin matrix had contributed to the effective enzyme action in the case of heptane medium although it is an extreme hydrophobic solvent. Pelts treated with 1% alpha-amylase in the three solvents showed better swelling compared to water medium. Increasing the enzyme concentration beyond 1% produced grain damage and loss of grain intactness in both control and experiments, which affects quality of the final leather. Hence, 1% enzyme concentration was chosen for further experiments.

### Fiber orientation and morphology of treated pelts

**Figure 5:**
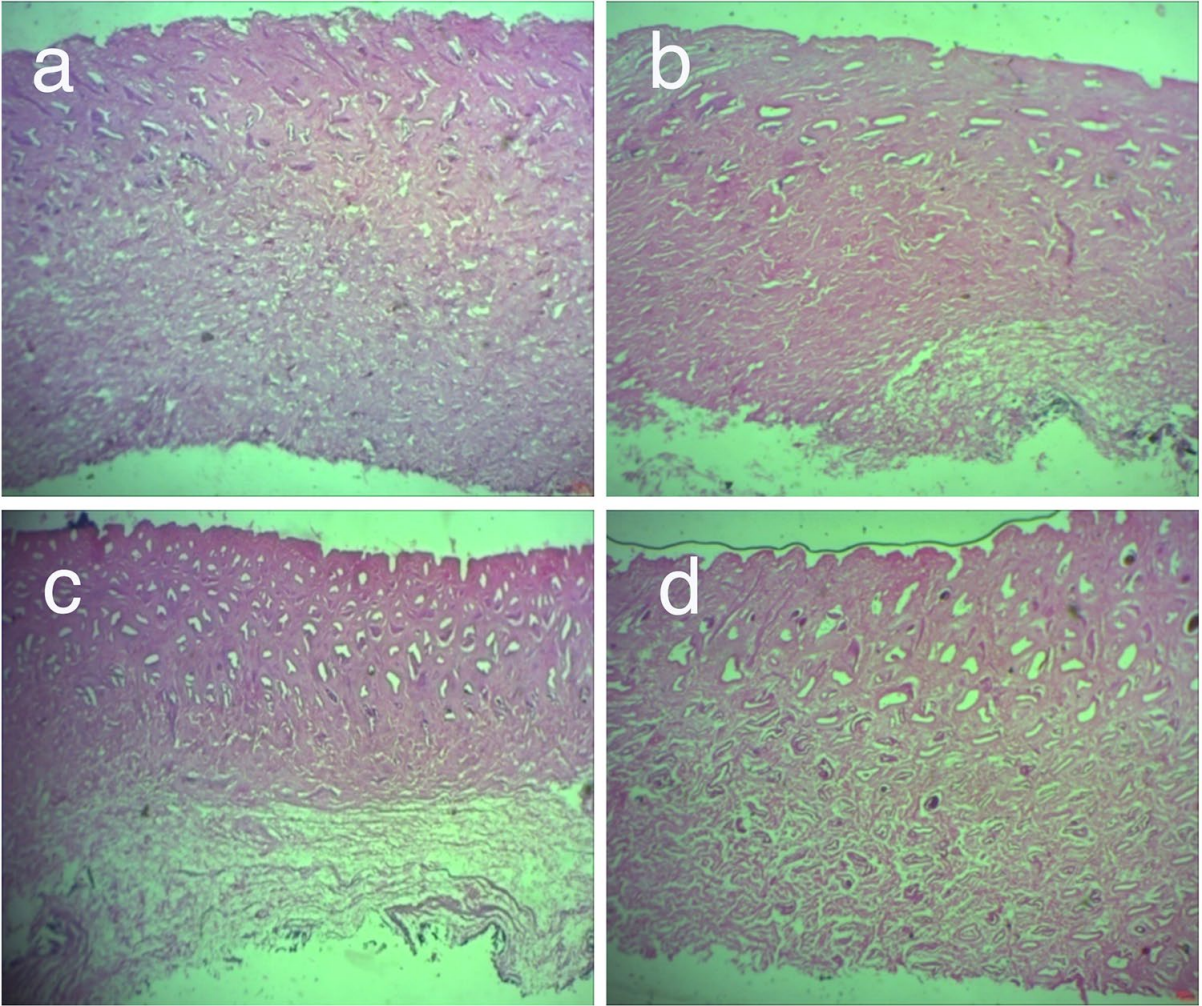
H&E stained sections of opened up sheep skins as viewed under a compound microscope at 100× magnification. (**a**) Control (water), (**b**) Heptane, (**c**) Propylene glycol and (**d**) PEG 200.

Histological analysis of the samples with H&E staining would provide an insight of the fiber opening at the tissue level. The stain, hematoxylin, shades mucin and chromatin as blue and eosin tints the cytoplasmic structures with varying shades of red to pink. The cytoplasm is scarcely colored^52^. Figure 5 (a–d) illustrates the H&E stained sections of opened up pelts from control and experimental processes. It is evident from the images that the compact structure of the fiber bundles has been loosened by alpha-amylase suggesting effective fiber opening. Opened pores on the top portion of the skin indicate effective removal of hair along with bulb during dehairing. The experimental pelts show comparable or even better degree of fiber opening to that of the control. Among the experimental solvents, PEG 200 was found to exhibit better fiber opening than the rest including control.

**Figure 6:**
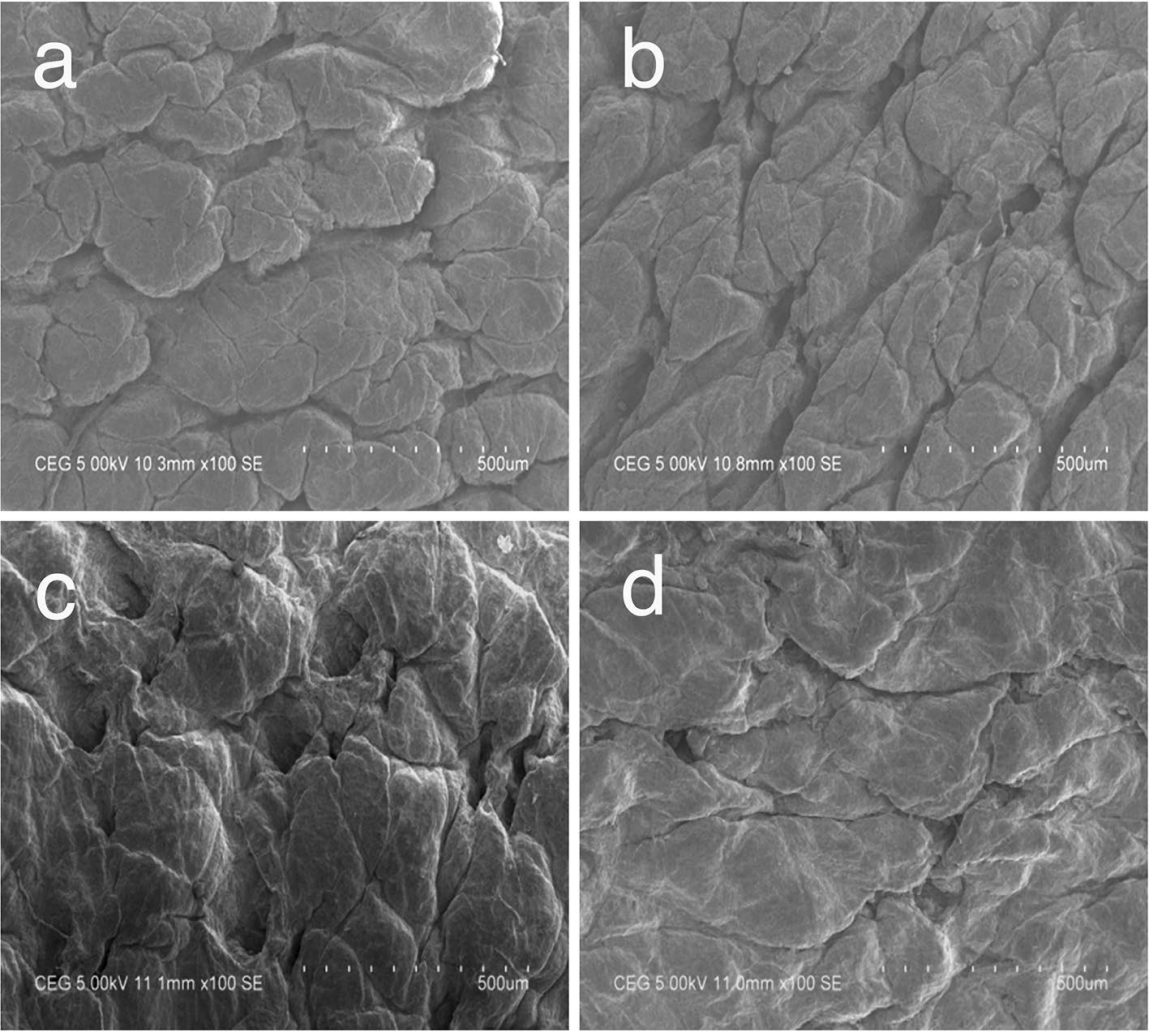
Scanning electron micrographs of grain surface of control and experimental leathers at 100× magnification (**a**) Control (water), (**b**) Heptane, (**c**) Propylene glycol and (**d**) PEG 200.

**Figure 7:**
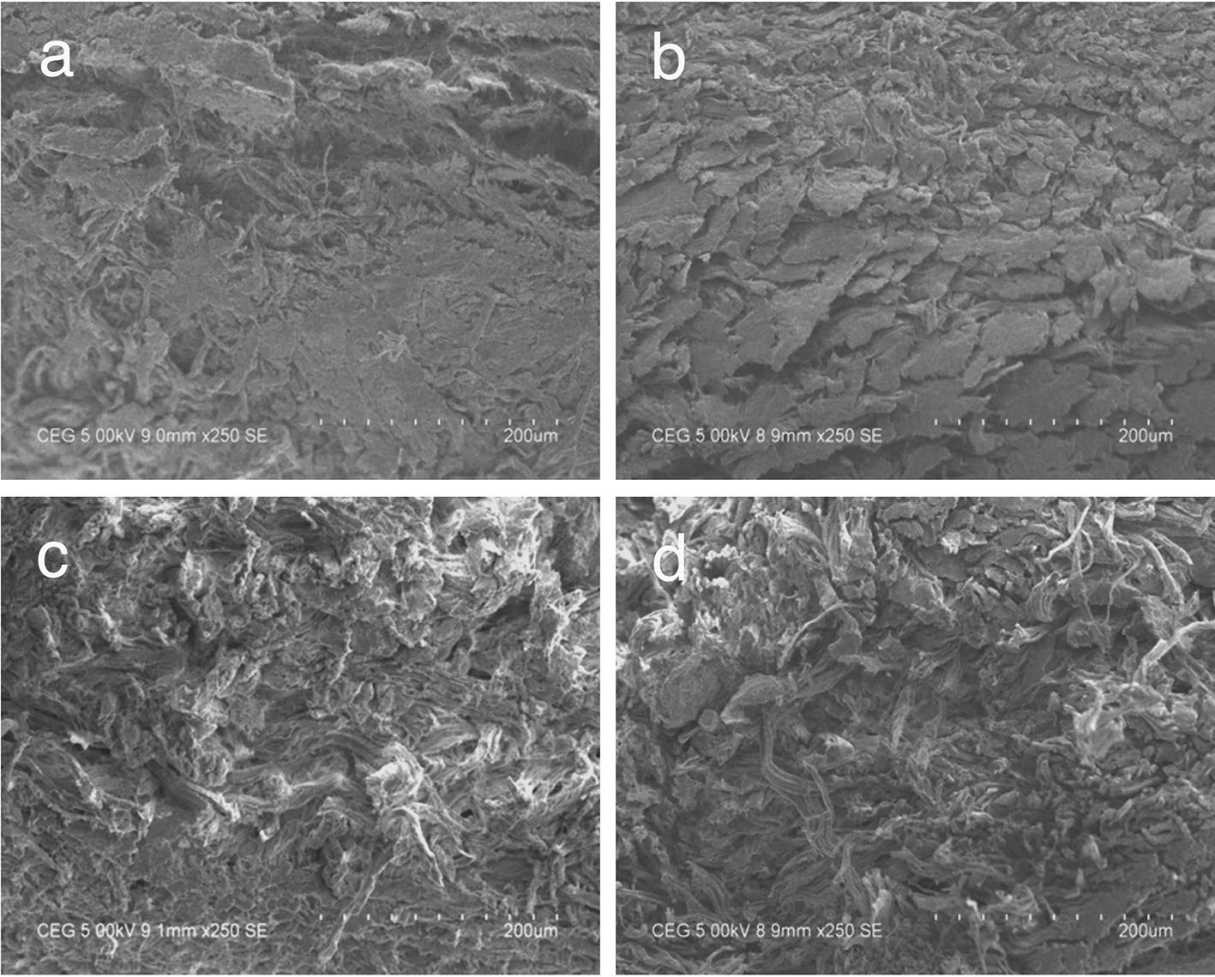
Scanning electron micrographs of cross section of control and experimental leathers at 250× magnification (**a**) Control (water), (**b**) Heptane, (**c**) Propylene glycol and (**d**) PEG 200.

Scanning electron micrographs of crust leather samples showing the surface and cross section from control and experiments are given in Figs. 6 (a–d) and 7 (a–d), respectively. The grain surfaces show similar pattern in control and all the experimental leathers. There is no detectable grain damage on the surface showing effective fiber opening and the enzyme applied for fiber opening did not rupture the grain. Cross sectional views indicate that the experimental leathers have higher degree of opening than control leather (water) suggesting efficient fiber opening in the green solvents than in water medium. Indeed, the fiber opening is found to be better in PEG 200 compared to other two solvents and water (control). This is in agreement with the data obtained in H&E staining as shown above.

### Shrinkage temperature of processed leathers

Shrinkage temperature was determined for experimental and control wet blue leathers. The shrinkage temperature is a measure of thermal stability of the wet blue leathers. Shrinkage temperature of the control leather was 103 ± 2 °C, while it was 101 ± 1 °C for heptane, 102 ± 2 °C for propylene glycol and 102 ± 1 °C for PEG 200 medium. Higher Ts values of above 100 °C for both experimental and control leathers indicate that the distribution of chromium is comparable due to the fairly analogous fiber opening and in turn providing stability to the samples processed in solvents. Therefore, the enzymatic fiber opening in solvents did not affect the thermal stability of the leathers.

### Physical and bulk properties of leathers

**Figure 8:**
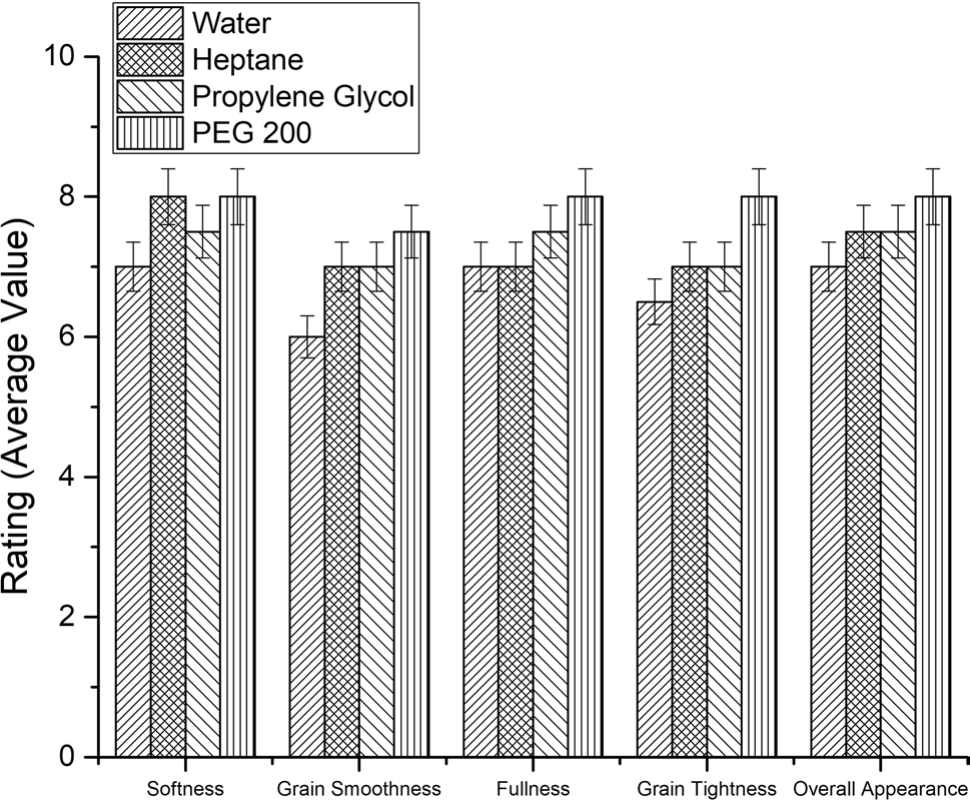
Organoleptic properties of control and experimental crust leathers.

Crust leathers prepared from both control and experiments were analyzed for physical and bulk properties. Tensile and tear strength testing along and across the backbone line were carried out and the mean values were calculated. Grain crack strength was measured using a lastometer and the mean was calculated. All the experimental leathers show comparable or even higher tensile strength, tear strength and grain crack strength than the control leathers (Table 1). Bulk properties such as softness, grain tightness, fullness, grain smoothness and overall appearance were assessed by experienced tanners. As can be seen from Fig. 8, all the bulk properties were comparable or better than the control leathers. Specifically, grain smoothness of experimental leathers was much better than control leathers. Among the three selected green solvents, PEG 200 resulted in leathers with outstanding bulk properties than leathers treated in other solvents as well as aqueous medium. Hence, it can be perceived that enzymatic fiber opening in green solvents does not impair the physical and bulk properties of the leathers.

**Table 1:** Physical properties of control and experimental leathers.

	Control	Heptane	Propylene glycol	PEG 200
Tensile strength (N/mm^2^)	16.1 ± 1.3	20.9 ± 1.5	18.0 ± 2.1	23.7 ± 1.8
Elongation at break (%)	67.3 ± 1.9	67 ± 2.5	60.3 ± 3.3	75.7 ± 4.2
Tear strength (N)	20.3 ± 0.5	20.9 ± 1.6	21.1 ± 0.8	22.4 ± 2.2
**Grain crack strength**				
Load (N)	210.7 ± 5.5	200.6 ± 8.2	230.2 ± 6.7	225.8 ± 9.1
Distention (mm)	8.9 ± 0.4	9 ± 0.7	9.3 ± 0.8	10.1 ± 0.5

### Environmental benefits

Spent liquor after each processing step from enzymatic fiber opening until tanning was collected separately and combined into a composite effluent according to the volume ratio. The composite liquor obtained was analyzed for TDS using the standard procedure. The concentration of TDS in the composite liquor and the emission load are presented in Table 2. It should be noted that no effluent was collected from the enzyme-only dehairing process for both control and experimental processes. Also, the enzymatic fiber opening process did not employ lime and hence the process of deliming is also eliminated in both control and experimental processes. As can be seen, TDS of composite liquor from experimental processes is lower than that of control process. This reduction in TDS concentration and emission load can be attributed to varying solubility of the compounds in the selected solvents and water. The fiber opening in solvent medium is expected to increase the levels of chemical oxygen demand than in control. This effect can be undone by recycling the used enzyme-solvent mixture for the subsequent trials, as shown in the following section.

**Table 2:** Composite liquor analysis for control and experimental leather processing.

Parameter	Control	Heptane	Propylene glycol	PEG 200
**Composite liquor**^#^				
TDS (mg/l)	35,620 ± 55	33,920 ± 82	30,960 ± 67	30,700 ± 61
Volume of effluent (L/t of raw skin)	3890	3820	3830	3830
**Emission load from composite liquor (kg/t of raw skin)**				
TDS	138.6	129.6	118.6	117.6

^#^Liquor collected up to chrome tanning excluding soak liquor.

### Reusability and stability of the spent solvent-enzyme mixture

**Figure 9:**
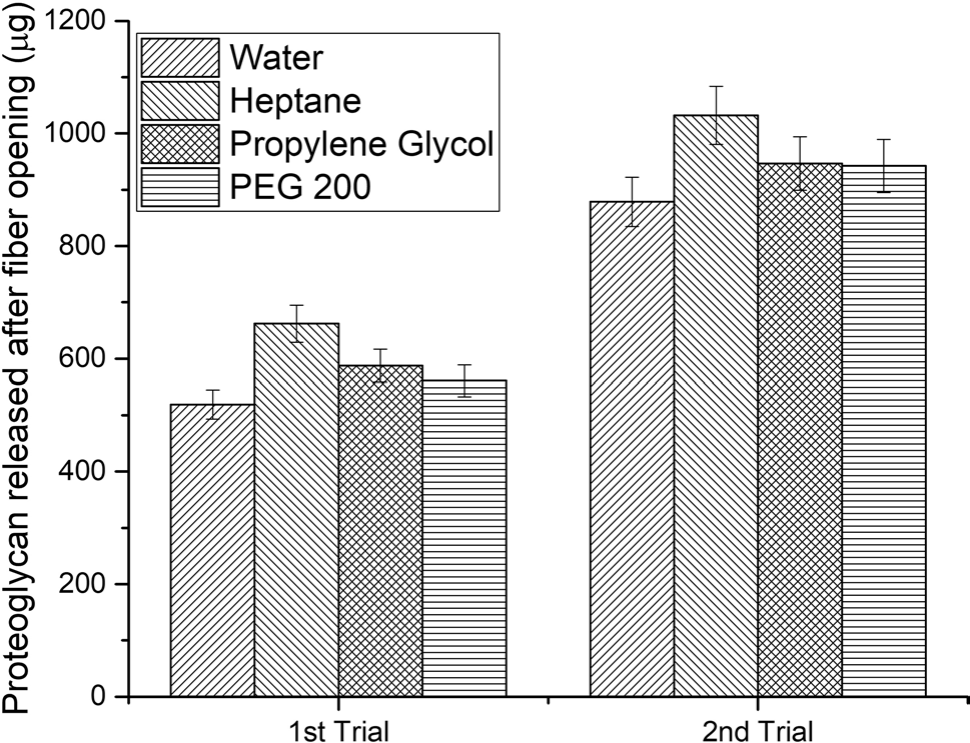
Proteoglycan release after fiber opening during recycling of solvent-enzyme mixture.

The solvent-enzyme mixture used for fiber opening of skins in the first trial was recovered and reused with no further addition of enzyme. There was up to 10% reduction in the volume of the solvent after recovery after the first batch of fiber opening process, which was supplemented with fresh solvent (Table 3). The efficiency of enzyme was validated by quantifying the extent of fiber opening in terms of proteoglycan release after each recycling trial. Fiber opening is the process of removal of non-collagenous cementing substances such as proteoglycans that reinforce the collagen fiber bundles. Removal of these cementing substances splits the fiber bundles into individual fibers and then into fibril level. Fiber opening process ensures better crosslinking of the collagen at fibril level during the subsequent chrome tanning process. Hence, the extent of fiber opening can be quantitatively analyzed by measuring the release of proteoglycans after the fiber opening process. The proteoglycan concentration in the fiber opening liquor was estimated using Schiff’s assay. The process liquor was filtered and subjected to spectrophotometric analysis. In the case of the solvent treated liquors, respective solvent blank was used. From the assay, it is seen that the release of proteoglycan is higher in all the experimental solvents compared to control (Fig. 9). Heptane showed highest release of proteoglycan among the chosen experimental solvents followed by propylene glycol and PEG 200. This can be correlated with the high activity of the enzyme in heptane medium (Fig. 1). The proteoglycan release is also high in the reused mixture for all the three solvents indicating efficient fiber opening even during recycling.

**Table 3:** Recycling of the used solvent-enzyme mixture in the fiber opening process.

Parameters	Water	Heptane	Propylene glycol	PEG 200
Solvent recovered in trial 1 (%)	85.2	91.5	96.1	96
Solvent recovered in trial 2 (%)	75.7	85	90.4	90
Residual enzyme activity (mmol/min/ml) in trial 1	4.07	6.32	4.51	5.88
Residual enzyme activity (mmol/min/ml) in trial 2	3.17	4.95	3.72	4.35

The residual activity of alpha-amylase in used solvent-enzyme mixture up to two consecutive trials was studied. It is observed that alpha-amylase retained around 70% activity in each solvent after the reuse (Table 3). Propylene glycol showed highest residual activity, 80%, among other solvents. The enzyme activity values of all the solvents are higher than that of water after the reuse. Hence, it should be noted that no additional alpha-amylase was used for the 2nd trial during recycling. Recycling of the solvent-enzyme mixture reduces the environmental and cost burden levied by them. Further, we also analyzed the activity of alpha-amylase in the solvent-enzyme mixture after 30 days and found that the enzyme retained its activity (5.88, 5.17 and 4.78 mmol/min/ml in heptane, PEG 200, propylene glycol, respectively). Therefore, the stability and recyclability of alpha-amylase in the selected green solvents offer great potential for application in fiber opening of skins/hides during leather processing.

## Conclusions

The global impact on environmental issues has become a serious concern to all industries including leather; hence the shift from conventional to green technologies is an essential factor for their growth and sustainability. This investigation has attempted to provide a green approach for reduction of chemicals as well as water for the processing of sheep skins. Water was replaced with non-aqueous green solvents in alpha-amylase based fiber opening process for improved process efficiency as well. Out of the seven green solvents, heptane, propylene glycol and PEG 200 were chosen for fiber opening of skins based on activity and kinetics data. In trials on optimization of fiber opening, it was found that 1% alpha-amylase was sufficient to bring about an efficient fiber opening comparable to the control without any grain damage. Morphological, bulk and physical property analysis of both control and experimental leathers show that the extent of fiber opening is satisfactory with a clean grain surface and the quality of the leather is not affected. Further, it is shown that the recycling of the solvent-enzyme mixture is feasible thereby suggesting a possible application of solvents in enzymatic processes industrially.

## Supplementary information

Supplementary Information 1.
