# Supplementary material for: Non-aqueous green solvents improve alpha-amylase induced fiber opening in leather processing
Source: Sci Rep. 2020 Dec 17;10:22274. doi: 10.1038/s41598-020-79406-8 (PMC7746733; doi:10.1038/s41598-020-79406-8)
Supplement: Supplementary file 1 — Supplementary Information 1. [file 41598_2020_79406_MOESM1_ESM.docx]

**Supplementary Information**

**Non-Aqueous Green Solvents Improve Alpha-Amylase Induced Fiber Opening in Leather Processing**

**Poornima Ramamoorthi^1^, Aravindhan Rathinam^1^*, Raghava Rao Jonnalagadda^2^, Thanikaivelan Palanisamy^3^***

^1^Leather Processing Technology Division, CSIR-Central Leather Research Institute, Adyar, Chennai 600020, India.

^2^Inorganic and physical chemistry laboratory, CSIR-Central Leather Research Institute, Adyar, Chennai 600020, India.

^3^Advanced Materials Laboratory, CSIR-Central Leather Research Institute, Adyar, Chennai 600020, India.

*Corresponding authors

Central Leather Research Institute (Council of Scientific and Industrial Research), Adyar, Chennai 600020, India. Tel: +91 44 24437142.

*E-mail address*: thanik8@yahoo.com (P. Thanikaivelan); aravindhanclri@gmail.com (R. Aravindhan)


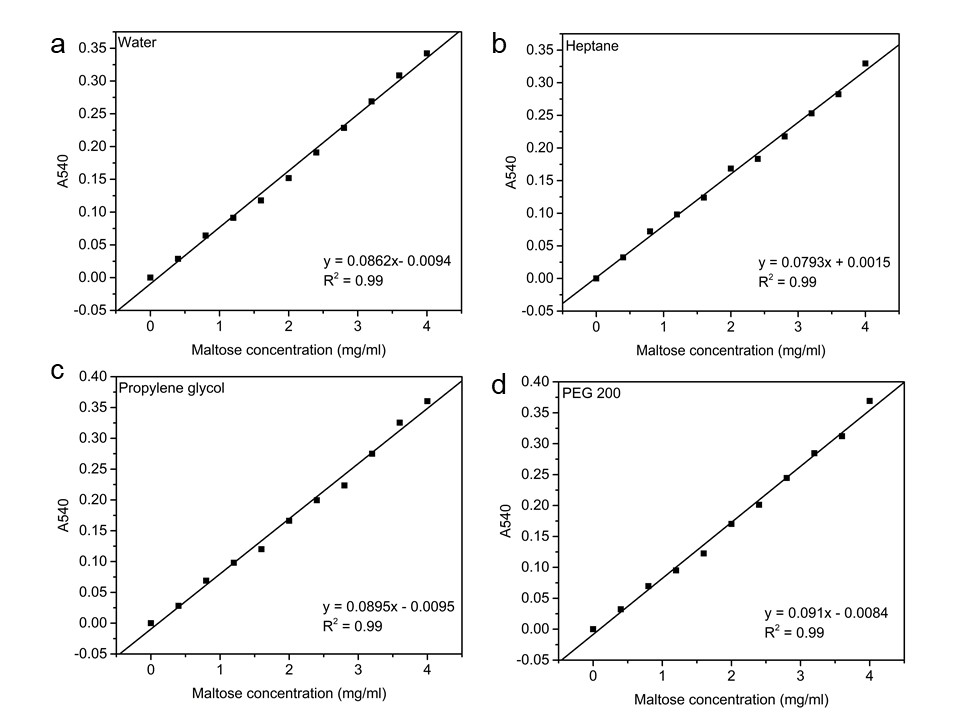


**Figure S1.** Standard graph of A540 vs [Maltose] in the presence of solvents such as (a) Water, (b) Heptane, (c) Propylene glycol and (d) PEG 200.


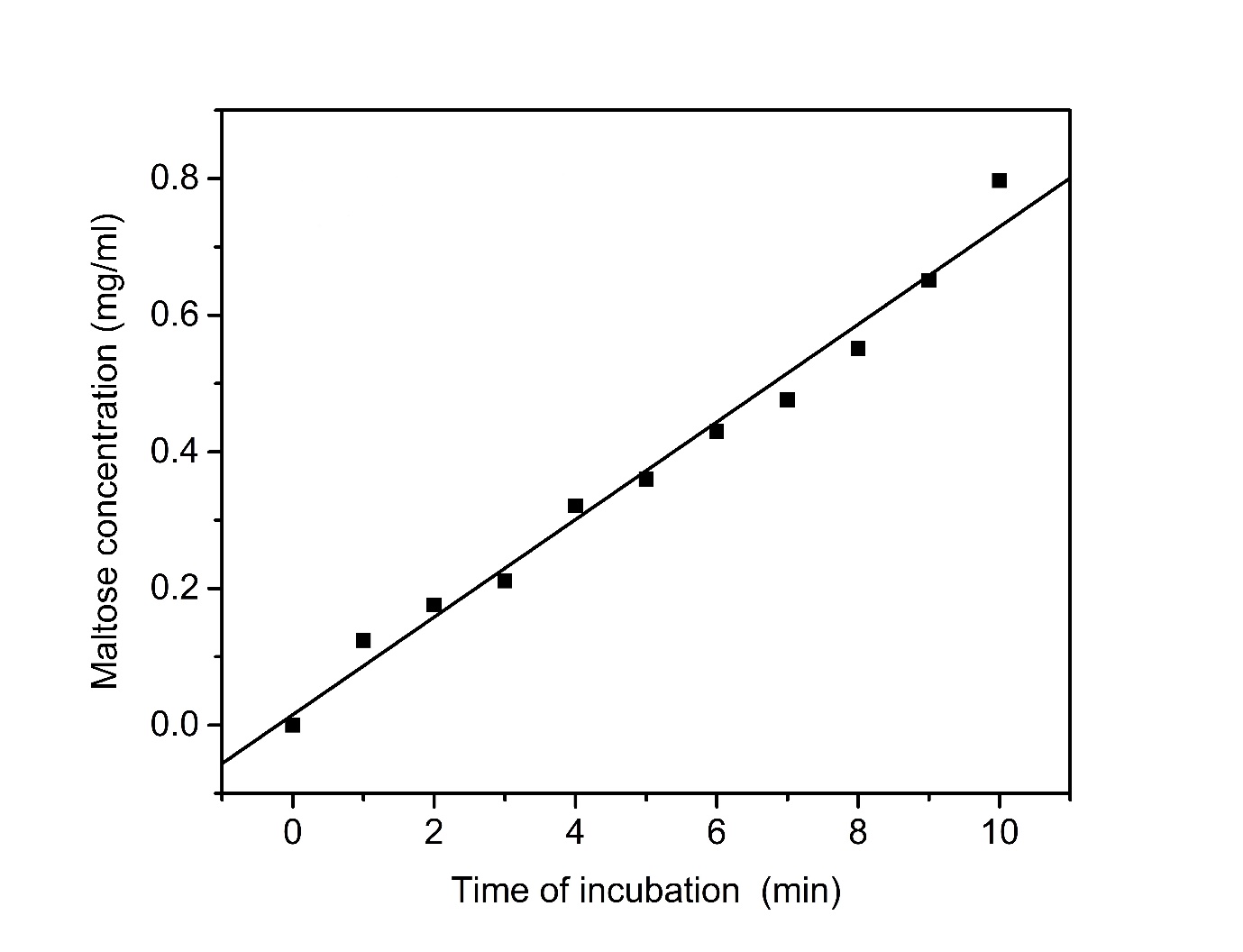


**Figure S2.** Concentration of maltose released with time of incubation.

The end point assay at the specified reaction conditions i.e., at pH 6.9, 30℃, 1 ml starch concentration of 10 mg/ml and enzyme volume of 1 ml (50 mg/ml), was carried out from 1 to 10 min of incubation (Fig. S2). As can be seen, the initial reaction rates followed linearity over a period of 10 min.

**Table S1 (a-e)** shows the raw data for determining the enzyme reaction rate. (a) Activity of the enzyme in various green solvents; (b-e) Enzyme kinetics in Water, Heptane, Propylene Glycol and PEG 200 respectively

(a) Activity of the enzyme in various green solvents

| Solvent | Abs 540 | Mg of maltose | mmol of maltose released | Activity (mmol/ml/min) |
| --- | --- | --- | --- | --- |
| Ethanol | 0.082 | 1.060 | 3.097 | 4.02 |
| Heptane | 0.1382 | 1.71 | 5 | 6.5 |
| PG | 0.111 | 1.39 | 4.08 | 5.3 |
| PEG 200 | 0.127 | 1.58 | 4.62 | 6 |
| Propylene carbonate | 0.0602 | 0.807 | 2.36 | 3.07 |
| Ethyl acetate | 0.0498 | 0.687 | 2 | 2.61 |
| Ethyl lactate | - | - | - | - |
| Water | 0.095 | 1.21 | 3.54 | 4.6 |

(b) Enzyme kinetics data in water

| Starch Concentration (mg/ml) | Abs Avg 540 | mg of maltose | mmol of maltose | Activity  (mmol/min/ml)  V | 1/V  (ml.min/mmol) | 1/S  (ml/mg) |
| --- | --- | --- | --- | --- | --- | --- |
| 0 | 0 | 0 | 0 | 0 | 0 | 0 |
| 10 | 0.139 | 1.72 | 5.03 | 6.54 | 0.153 | 0.1 |
| 20 | 0.1998 | 2.43 | 7.09 | 9.22 | 0.108 | 0.05 |
| 30 | 0.2334 | 2.82 | 8.23 | 10.69 | 0.093 | 0.033 |
| 40 | 0.3038 | 3.63 | 10.61 | 13.8 | 0.072 | 0.025 |
| 50 | 0.3134 | 3.74 | 10.94 | 14.22 | 0.070 | 0.02 |
| 60 | 0.3158 | 3.77 | 11.02 | 14.32 | 0.0697 | 0.0167 |
| 70 | 0.3174 | 3.79 | 11.07 | 14.4 | 0.0694 | 0.0143 |
| 80 | 0.3142 | 3.75 | 10.97 | 14.26 | 0.07 | 0.0125 |
| 90 | 0.3158 | 3.77 | 11.02 | 14.33 | 0.0697 | 0.011 |
| 100 | 0.3166 | 3.78 | 11.05 | 14.36 | 0.0696 | 0.01 |

| Starch Concentration (mg/ml) | Abs Avg 540 | mg of maltose | mmol of maltose | Activity  (mmol/min/ml)  V | 1/V  (ml.min/mmol) | 1/S  (ml/mg) |
| --- | --- | --- | --- | --- | --- | --- |
| 0 | 0 | 0 | 0 | 0 | 0 | 0 |
| 10 | 0.1338 | 1.66 | 4.85 | 6.31 | 0.158 | 0.1 |
| 20 | 0.1966 | 2.39 | 6.98 | 9.1 | 0.11 | 0.05 |
| 30 | 0.2262 | 2.73 | 7.98 | 10.38 | 0.096 | 0.033 |
| 40 | 0.255 | 3.07 | 8.96 | 11.65 | 0.086 | 0.025 |
| 50 | 0.2714 | 3.26 | 9.52 | 12.37 | 0.081 | 0.02 |
| 60 | 0.271 | 3.25 | 9.5 | 12.35 | 0.081 | 0.0167 |
| 70 | 0.2756 | 3.31 | 9.66 | 12.56 | 0.08 | 0.0143 |
| 80 | 0.275 | 3.3 | 9.64 | 12.53 | 0.079 | 0.0125 |
| 90 | 0.276 | 3.31 | 9.67 | 12.57 | 0.0795 | 0.011 |
| 100 | 0.2766 | 3.32 | 9.69 | 12.6 | 0.0793 | 0.01 |

| Starch Concentration (mg/ml) | Abs Avg 540 | mg of maltose | mmol of maltose | Activity  (mmol/min/ml)  V | 1/V  (ml.min/mmol) | 1/S  (ml/mg) |
| --- | --- | --- | --- | --- | --- | --- |
| 0 | 0 | 0 | 0 | 0 | 0 | 0 |
| 10 | 0.1094 | 1.38 | 4.03 | 5.23 | 0.191 | 0.1 |
| 20 | 0.1854 | 2.26 | 6.6 | 8.58 | 0.116 | 0.05 |
| 30 | 0.343 | 4.09 | 11.94 | 15.53 | 0.064 | 0.033 |
| 40 | 0.3318 | 3.96 | 11.56 | 15.03 | 0.066 | 0.025 |
| 50 | 0.3320 | 3.96 | 11.57 | 15.04 | 0.066 | 0.02 |
| 60 | 0.3319 | 3.96 | 11.57 | 15.04 | 0.066 | 0.0167 |
| 70 | 0.333 | 3.97 | 11.6 | 15.08 | 0.066 | 0.0143 |
| 80 | 0.332 | 3.96 | 11.57 | 15.04 | 0.0665 | 0.0125 |
| 90 | 0.3342 | 3.99 | 11.64 | 15.14 | 0.0661 | 0.011 |
| 100 | 0.334 | 3.98 | 11.63 | 15.13 | 0.0661 | 0.01 |

(c) Enzyme kinetics data in heptane

(d) Enzyme kinetics data in propylene glycol

(e) Enzyme kinetics data in PEG 200

| Starch Concentration (mg/ml) | Abs Avg 540 | mg of maltose | mmol of maltose | Activity  (mmol/min/ml)  V | 1/V  (ml.min/mmol) | 1/S  (ml/mg) |
| --- | --- | --- | --- | --- | --- | --- |
| 0 | 0 | 0 | 0 | 0 | 0 | 0 |
| 10 | 0.1374 | 1.7 | 4.98 | 6.46 | 0.155 | 0.1 |
| 20 | 0.307 | 3.67 | 10.72 | 13.94 | 0.072 | 0.05 |
| 30 | 0.267 | 3.21 | 9.37 | 12.18 | 0.082 | 0.033 |
| 40 | 0.3246 | 3.87 | 11.32 | 14.72 | 0.068 | 0.025 |
| 50 | 0.3214 | 3.84 | 11.21 | 14.57 | 0.069 | 0.02 |
| 60 | 0.3216 | 3.84 | 11.21 | 14.57 | 0.069 | 0.0167 |
| 70 | 0.3212 | 3.84 | 11.21 | 14.57 | 0.069 | 0.0143 |
| 80 | 0.323 | 3.86 | 11.26 | 14.64 | 0.0683 | 0.0125 |
| 90 | 0.3234 | 3.86 | 11.26 | 14.64 | 0.0683 | 0.011 |
| 100 | 0.3238 | 3.865 | 11.29 | 14.68 | 0.0681 | 0.01 |

Enzyme activity was calculated using the following formula

$Enzyme activity \left( mmol/min/ml \right)=\frac{\left[ Maltose \right] X Dilution factor}{Volume of enzyme used X time of assay X MW of maltose}$

Where,

[Maltose] = Concentration of maltose obtained from standard graph in mg/ml,

Dilution factor = Total volume used in the assay, ml = 13 ml

Volume of enzyme used = 1ml

Time of assay = Total time of incubation substrate with the enzyme = 10 min

MW of maltose = Molecular weight of maltose = 342.3 g/mol

**Table S2.** Leather processing procedure for experimental and control crust leathers

| **Process** | **Chemicals** | | **% offer** | | **Time (min)** | **Remarks** | |  |
| --- | --- | --- | --- | --- | --- | --- | --- | --- |
| After the enzymatic fiber opening process, the pelts were defleshed using fleshing machine. | | | | | | | |  |
| Washing | Water | | 100 | | 10 | Drained | |  |
| Pickling | Water | | 100 | |  |  | |  |
|  | Sodium chloride | | 10 | | 10 | Checked salt concentration and found to be 9^o^Be | |  |
|  | Sulphuric acid | | 1 | | 3x15+60 | pH 2.8-3.0; drained 50% of the float | |  |
| Chrome tanning | Pickle float | | 50 | |  |  | |  |
|  | Basic chromium sulphate | | 8 | | 2 x 30 + 1 h |  | |  |
|  | Water | | 50 | | 30 | Checked for complete penetration | |  |
| Basification | Sodium formate | | 1.5 | | 10 |  | |  |
|  | Sodium bicarbonate | | 0.5 | | 3 x 15 + 1 h |  | |  |
| pH adjusted to 3.8-4.0, drained, washed and the skins were piled for 2 days. | | | | | | | |  |
| Washing | Water | 100 | | 10 | | | Drained | |
| Neutralization | Water  Neutralization syntan | 150  1.5 | | 20 | | | Drained  pH = 5.5±0.1 | |
| Retanning, Dyeing & Fat liquoring | Water  Acrylic syntan  Phenolic syntan  Vegetable syntan  Acid dye  Sulphited fatliquor  Lecithin based fatliquor  Lanolin based fatliquor  Phenolic syntan  Formic acid | 80  2  5  5  3  3  3  2  2  2 | | 20  45  30  45  20  3 x 10 + 45 | | | Checked for complete penetration  Drained and washed | |

Leathers were piled overnight. Next day, leathers were set, dried, staked and buffed to obtain crust leathers.
